# Hydrogen Bond‐Mediated Self‐Shielded Moisture‐Responsive Structural Color for Time‐Temperature Indicating

**DOI:** 10.1002/advs.202310060

**Published:** 2024-02-26

**Authors:** Donghui Kou, Lei Gao, Ruicheng Lin, Shufen Zhang, Wei Ma

**Affiliations:** ^1^ State Key Laboratory of Fine Chemicals Frontier Science Center for Smart Materials Dalian University of Technology Dalian 116024 China

**Keywords:** hydrogen bonds, photonic crystals, responsiveness, structural colors, time‐temperature indicators

## Abstract

Effective monitoring of the time‐temperature history of biological reagents, chemical drugs, and perishable foods during cold chain storage is crucial for ensuring their quality and efficacy. Time‐temperature indicators (TTIs) are developed to assess the cumulative impact of time and temperature on product quality. However, current indicators face challenges related to complex wrapping procedures, narrow tracking ranges, susceptibility to photobleaching, and pre‐use instability, hampering widespread use. Herein, the first moisture‐responsive 1D photonic crystal (1DPC) TTIs featuring robust structural colors, customizable time‐temperature ranges, and reliable renewability are demonstrated. The indicators exhibit distinct color‐changing responsiveness toward water vapor, which remains observable after prolonged storage at low temperatures. Significantly, the moisture responsiveness gradually diminishes at elevated temperatures over time due to ambient water‐induced hydrogen bond formation, effectively shielding the indicator from external stimuli. This property enables the naked‐eye inspection of product efficacy during cold chain storage. Additionally, the endowed flexibility of the TTI facilitates its easy attachment to targets, functioning as a convenient indicator label. Remarkably, the indicator can be stably stored for an extended period at room temperature before use, thereby showcasing substantial market potential.

## Graphical abstract

A versatile reusable photonic crystal‐based time‐temperature indicator with self‐shielded responsive structural color is developed for perishable products in cold chain logistics. The indicator presents a wide tracking temperature range, customizable monitoring time, and high pre‐use stability. It can also serve as both a smart anti‐counterfeit label and a hygroscopic indicator for moisture‐sensitive materials.

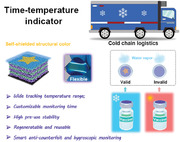

## 1 Introduction

In contemporary society, cold chain logistics has emerged as a crucial conduit for safeguarding livelihoods and connecting global fresh food supplies.^[^ ^1^ ^]^ An uninterrupted series of processes, equipment, and protocols have been designed to uphold low temperatures, ensuring the secure transportation and storage of temperature‐sensitive products.^[^ ^2^ ^]^ Inadequate temperature regulation during storage can result in a decline in product quality. Typically, biomaterials, such as stem cells, enzymes, vaccines, and blood necessitate low‐temperature storage to ensure safety prior to use, otherwise will rapidly deteriorate.^[^ ^3^ ^]^ For perishable foods, prolonged temperature elevation accelerates the growth of pathogens and spoilage microorganisms.^[^ ^4^ ^]^ In a targeted manner, time‐temperature indicators (TTIs) were designed to assess the cumulative impact of time‐temperature history on product quality.^[^ ^5^ ^]^ The essential criteria for a TTI include displaying continuous irreversible changes, temperature‐dependent escalating change rate, high pre‐use stability, and easy detectability.^[^ ^6^ ^]^

Electronic TTIs provide high monitoring precision and accuracy, but suffer from high costs and electronic waste.^[^ ^7^ ^]^ Material‐based TTIs, with active additives like chemicals or dyes, offer visual data on time‐temperature history and are promising waste‐free alternatives.^[^ ^8^ ^]^ Material‐derived TTIs can be broadly classified into two types: chemical‐reaction‐activated and diffusion‐based.^[^ ^9^ ^]^ The former relies on color changes initiated by chemical reactions involving an enzyme or petrochemical, activated at a specific temperature threshold.^[^ ^10^ ^]^ However, these types of TTIs are generally activated at elevated temperatures, rendering them unsuitable for products requiring subzero storage. Moreover, the potential risk of leakage of reactive additives also exists, posing a safety hazard.^[^ ^9^ ^]^ Dye‐diffusion‐activated TTIs can function at lower temperatures. They employ a temperature‐sensitive dye and porous material, with the dye undergoing phase change and permeating the membrane above its melting point.^[^ ^11^ ^]^ Whereas, these TTIs also exhibit notable drawbacks such as susceptibility to photobleaching and a limited tracking temperature range. Additionally, they require a rigid casing for modularization, increasing bulk and cost.^[^ ^12^ ^]^ Hence, there is a pressing demand for advanced materials possessing stability, sensitivity, and a broad temperature range to ensure the reliability of smart TTIs.

Structural color materials, like photonic crystals (PCs),^[^ ^13^ ^]^ interference films,^[^ ^14^ ^]^ diffraction gratings,^[^ ^15^ ^]^ and wrinkles,^[^ ^16^ ^]^ are emerging as smart, bioinspired materials. Their colors arise from the interactions between visible light and periodic structures, showcasing remarkable resistance to photobleaching.^[^ ^17^ ^]^ PCs, particularly responsive ones, are extensively explored for displays,^[^ ^18^ ^]^ optical devices,^[^ ^19^ ^]^ and chemical/biosensors.^[^ ^20^ ^]^ According to their periodic distribution in spatial dimensions, PCs can be classified into 1D, 2D, and 3D structures.^[^ ^21^ ^]^ For pursuing smart TTIs, diverse time‐dependent thermal‐responsive PCs have been designed.^[^ ^22^ ^]^ These materials demonstrated swift and irreversible color changes upon reaching temperatures beyond their glass transition temperature or lower critical solution temperature. However, the critical temperature for continuous color changes in PCs exceeded 0 °C and even higher than room temperature, making them unsuitable for monitoring time‐temperature dynamics in low‐temperature environments. Recently, a self‐destructive structural color liquid (SCL), composed of a triggering agent and liquid colloidal PC, was proposed for TTI.^[^ ^23^ ^]^ The SCL showed intrinsic irreversibility, tunable self‐destructive time, and a broad temperature tracking range (−70 to +37 °C). Whereas, the preparation period of this liquid colloidal PC is prolonged, and it is challenging to store for an extended period at room temperature before use.

**Figure 1:**
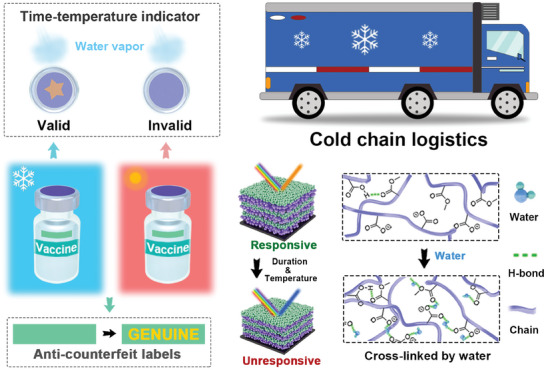
Schematic diagram of the TTIs for assessing the cumulative impact of time‐temperature history on product quality and application as a smart anti‐counterfeit label.

To tackle these challenges, we developed an innovative TTI employing a moisture‐responsive 1DPC (**Figure** **1**). The indicator was facilely fabricated by stacking carboxyl‐containing copolymer and TiO_2_ layers onto a substrate, followed by alkaline treatment for carboxylate anion introduction. Due to the pronounced affinity of carboxylate ions for water molecules, the 1DPC presents a heightened and responsive color change in response to water vapor. This feature remained effective for an extended duration at low temperatures. The color‐changing responsiveness is lost at elevated temperatures over time owing to ambient water‐induced hydrogen bond formation, locking the indicator into a self‐shielded state, and resisting external moisture stimuli. The TTI enables a straightforward visual assessment of product efficacy during cold chain storage, offering high sensitivity, customizable time, and broad temperature monitoring ranges. Significantly, by isolating ambient moisture, the TTI can be stored at room temperature for extended periods prior to use. With a flexible substrate, the TTI can be conveniently affixed to objects with diverse shapes. Beyond its primary purpose, the indicator's unique responsiveness makes it a promising candidate for anti‐counterfeit applications and enables visualization of moisture absorption of hygroscopic reagents during dry storage.

## 2 Results and Discussion

### 2.1 Fabrication of 1DPC‐Based TTIs

**Figure 2:**
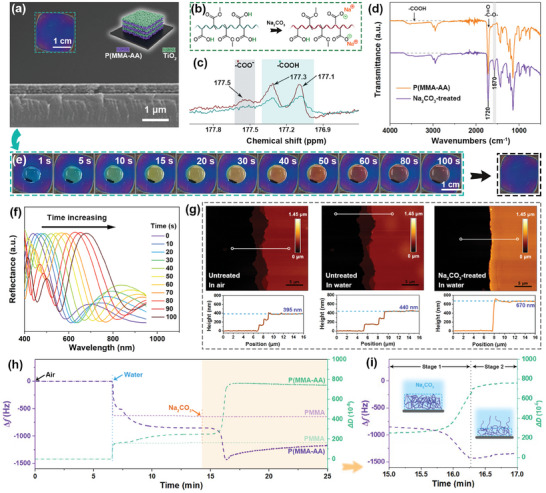
a) Photograph, structural schematic diagram, and cross‐sectional SEM image of the 1DPC. b) Schematic diagram of molecular formula, c) ^13^C NMR spectra, and d) FTIR spectra of P(MMA‐AA) before and after Na_2_CO_3_ treatment. e) Photographs and f) reflection spectra of the 1DPC exposed to 0.1 m Na_2_CO_3_ solution. g) 3D AFM images and height profiles of the 1DPC measured in air and water before and after Na_2_CO_3_ treatment. h) Frequency and dissipation changes of P(MMA‐AA) and PMMA films exposed to air, water, and Na_2_CO_3_ solution. i) Enlarged image of Figure h and schematic diagram of P(MMA‐AA) in Na_2_CO_3_ solution.

To fabricate 1DPC‐based TTIs, carboxyl‐containing yet hydrophobic poly(methyl methacrylate‐acrylic acid) (P(MMA‐AA)) copolymer was initially designed, and shaped into nanoparticles for easy assembly (Figure S1, Supporting Information). PMMA, as the predominant copolymer component, plays a dual role in preserving the hydrophobicity of 1DPC and offering ample oxygen‐containing groups that serve as sites for hydrogen bond formation. The carboxyl groups within PAA, acting as active moieties, are utilized to modulate the subsequent formation and dissociation of hydrogen bonds. We identified the optimal copolymer composition with a PMMA to PAA ratio of approximately 12:1, as confirmed by ^1^H NMR spectrum analysis (see Experimental Section and Figure S2, Supporting Information). Subsequently, a yellow three‐stacked P(MMA‐AA)/TiO_2_ 1DPC was fabricated on a silicon wafer via a spin‐coating technique (Figure S3, Supporting Information). To enhance structural stability, the 1DPCs underwent sequential treatments of ethanol vapor and heat, concurrently declining the copolymer's thickness (details see Experimental Section and Figure S4, Supporting Information). Consequently, a purple 1DPC was achieved, featuring thicknesses of copolymer and TiO_2_ layers of 60 and 75 nm, respectively (**Figure** **2**a).

Then, the central areas of the 1DPCs were treated with Na_2_CO_3_ aqueous solutions to convert ─COOH to ─COO^−^ (Figure 2b). After treatment of Na_2_CO_3_ solution, a new peak of 177.5 ppm assigned to ─COO^−^ of the copolymer appeared in ^13^C NMR spectra (Figure 2c). The FTIR spectra of Figure 2d present a characterized peak at 1570 cm^−1^, confirming the formation of carboxylate anion. Visually, after 0.1 m Na_2_CO_3_ solution was added, the color of the central pattern on the photonic film varied from violet to dark red within 100 s, spanning the entire visible light wavelength range (Figure 2e). Accordingly, the stopband wavelength shifted from 445 to 680 nm (Figure 2f). When the Na_2_CO_3_ solution was removed and the photonic film was dried, the central color reverted to violet (Figure 2e; Figure S5, Supporting Information). Notably, unless otherwise specified, the responsiveness, namely, the variations of color and wavelength in the following context refer to the Na_2_CO_3_‐treated pattern areas.

To investigate the color evolution mechanism, micro‐morphology variations of the 1DPC were characterized using an atomic force microscope (AFM). The thicknesses of the untreated 1DPC in air and water were measured to be 395 and 440 nm, respectively (Figure 2g). The color of the film changed from violet to blue as the wavelength shifted from 445 to 465 nm when immersed in water (Figure S6, Supporting Information). The results suggest the P(MMA‐AA) layers absorbed a small amount of water and swelled slightly. Remarkably, after being modified by Na_2_CO_3_, the thickness of the 1DPC in water increased to 670 nm. This is because, in Na_2_CO_3_ solutions, the P(MMA‐AA) underwent a transformation in which COO^−^ newly formed, resulting in an enhanced affinity of the copolymer for water. Consequently, the P(MMA‐AA) exhibited a pronounced swelling behavior in water, leading to a significant increase in thickness and hue changes of structural color.

Further, quartz crystal microbalance with dissipation monitoring (QCM‐D) was employed to gain a comprehensive understanding of the changing process of the color and the microstructure of the photonic film (details see Experimental Section). As visualized in Figure 2h, the variations of frequency (Δ*f*) and dissipation (Δ*D*) of the PMMA and the P(MMA‐AA) films were monitored and compared. Above all, clean air passed through the test chamber, the recorded frequency and dissipation were set as the reference baselines (Δ*f* = 0; Δ*D* = 0). After deionized water was injected, the frequency of the PMMA film decreased and reached a balance (−623 Hz) within 15 s. Meanwhile, an increase in dissipation (160 ×10^−6^) could be observed. The observed fluctuations primarily resulted from the variations in fluid (air and water) density and viscosity. For the P(MMA‐AA) film, the frequency and the dissipation presented similar variation trends compared with that of the PMMA film at the first 15 s. The difference is that the frequency further declined slowly and reached the lowest value of −858 Hz after 6 min. The dissipation increased rapidly at first 15 s and then reached 250 × 10^−6^. The gradual changes in frequency and dissipation were attributed to the adsorption of water molecules and hydration‐induced swelling of P(MMA‐AA), owing to the presence of hydrophilic carboxyl groups.

Subsequently, 0.1 m Na_2_CO_3_ aqueous solutions were injected. No obvious changes in frequency and dissipation could be recognized for PMMA film. But as expected, the frequency continued to decrease (−1425 Hz) and the dissipation further increased (635 × 10^−6^) owing to the newly formed ─COO^−^ and its enhanced affinity for water molecules (stage 1, Figure 2i). Unusually, after the frequency decreased to its minimum value, it gradually increased thereafter, with dissipation consistently exhibiting an ascending trend (stage 2, Figure 2i). This may be attributed to the excessive swelling of the copolymer during this stage, wherein some copolymer chains extended into a fluid environment, liberating water molecules, reducing mass, and increasing frequency.

### 2.2 Moisture‐Responsive Structural Colors

**Figure 3:**
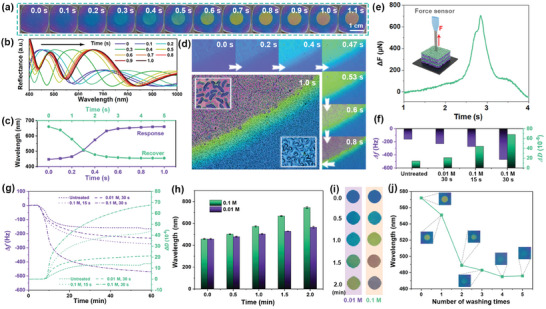
a) Photographs and b) reflection spectra of the 1DPC exposed to water vapor. c) Wavelength shifts during response and recovery processes. d) Optical microscope images of the 1DPC exposed to water vapor after treatment of Na_2_CO_3_ solution. e) Force changes of Na_2_CO_3_‐treated 1DPC exposed to water vapor. f,g) Frequency and dissipation changes of the copolymer film exposed to water vapor after being treated by Na_2_CO_3_ solutions with various conditions. h) Reflection wavelengths and i) photographs of the 1DPC exposed to water vapor after being treated by Na_2_CO_3_ solutions with various conditions. j) Variations of wavelength and color of the 1DPCs after washing with water at different times.

The generation of ─COO^−^ effectively enhanced the hydrophilicity of the P(MMA‐AA), evident in the reduced water contact angle from 63° to 42° (Figure S7, Supporting Information). Under water vapor stimulation, the Na_2_CO_3_‐treated copolymer layers in the 1DPCs absorbed more water and generated volumetric expansion, promoting significant wavelength redshifts and color variations. In **Figure** **3**a, upon exposure to water vapor from human breath, the Na_2_CO_3_‐treated pattern transformed rapidly from violet to red, reaching equilibrium in 1.0 s. Figure 3b presents distinct wavelength shifts from 445 to 660 nm. However, the untreated background only showed a subtle change from violet to blue. After water evaporation, the copolymer shrunk. Correspondingly, the wavelength of the 1DPC blue‐shifted (Figure 3c), the color reverted to violet and the pattern disappeared, presenting excellent responsiveness reversibility.

Optical microscope images in Figure 3d also recorded the response process. Notably, the temperature of exhaled water vapor exceeds the ambient temperature, leading to the rapid condensation of water droplets on the 1DPC upon contact. These water droplets could enhance the responsiveness of the 1DPCs. Additionally, the Na_2_CO_3_‐treated regions exhibited visible crack formations. These cracks served as effective pathways for water vapor permeation and diffusion, contributing to an accelerated response rate of the 1DPC. The responsive behavior of the 1DPCs was also proved using micro‐ and nano‐mechanical test systems (Figure 3e). When exposed to water vapor, the Δ*F* of the force sensor on the Na_2_CO_3_‐treated 1DPC increased to 705 µN, indicating a significant increase in thickness in the vertical direction. With the evaporation of water, the Δ*F* decreased to ≈0 immediately.

The ultimate responsive color of the 1DPC in water vapor can be modulated by adjusting Na_2_CO_3_ solution concentration or treatment time to alter carboxylate ion production. Figure 3f,g shows that the frequency of the untreated P(MMA‐AA) film decreased to −160 Hz upon exposure to water vapor and the dissipation increased to 13 × 10^−6^. After being treated with 0.01 m Na_2_CO_3_ solution for 30 s, the frequency and the dissipation in water vapor were measured to be −230 Hz and 21 × 10^−6^. For 0.1 m Na_2_CO_3_, when the treatment time was 15 and 30 s, the values were −270 Hz, 44 × 10^−6^ and −470 Hz, 68 × 10^−6^, respectively. The results indicate higher Na_2_CO_3_ concentrations and longer treatment time enhanced frequency and dissipation changes, improving responsiveness and swelling of the copolymer. From this, 1DPCs can be customized as a requirement for different water vapor responsiveness. As depicted in Figure 3h,i, treatment with 0.01 m and 0.1 m Na_2_CO_3_ for different durations resulted in 1DPCs displaying diverse colors with various wavelength locations toward water vapor.

Interestingly, the water vapor responsiveness of the 1DPCs can be temporarily concealed following repeated water washing. Figure 3j shows the responsive wavelength of the Na_2_CO_3_‐treated pattern in water vapor blue‐shifted with an increasing number of water washing cycles. Concurrently, the pattern weakened and almost disappeared after five washing cycles. As for the principle, alkaline treatment introduced residual Na_2_CO_3_ into the 1DPC. Upon exposure to water vapor, water droplets aggregated on the film, creating a weak alkaline environment. Due to osmotic pressure, more water molecules diffused into the 1DPC, leading to noticeable volume expansion and color changes. While subsequent water washes resulted in a reduction of residual Na_2_CO_3_, gradually diminishing the film's responsiveness to water vapor.

### 2.3 Self‐Shielded Behaviors

**Figure 4:**
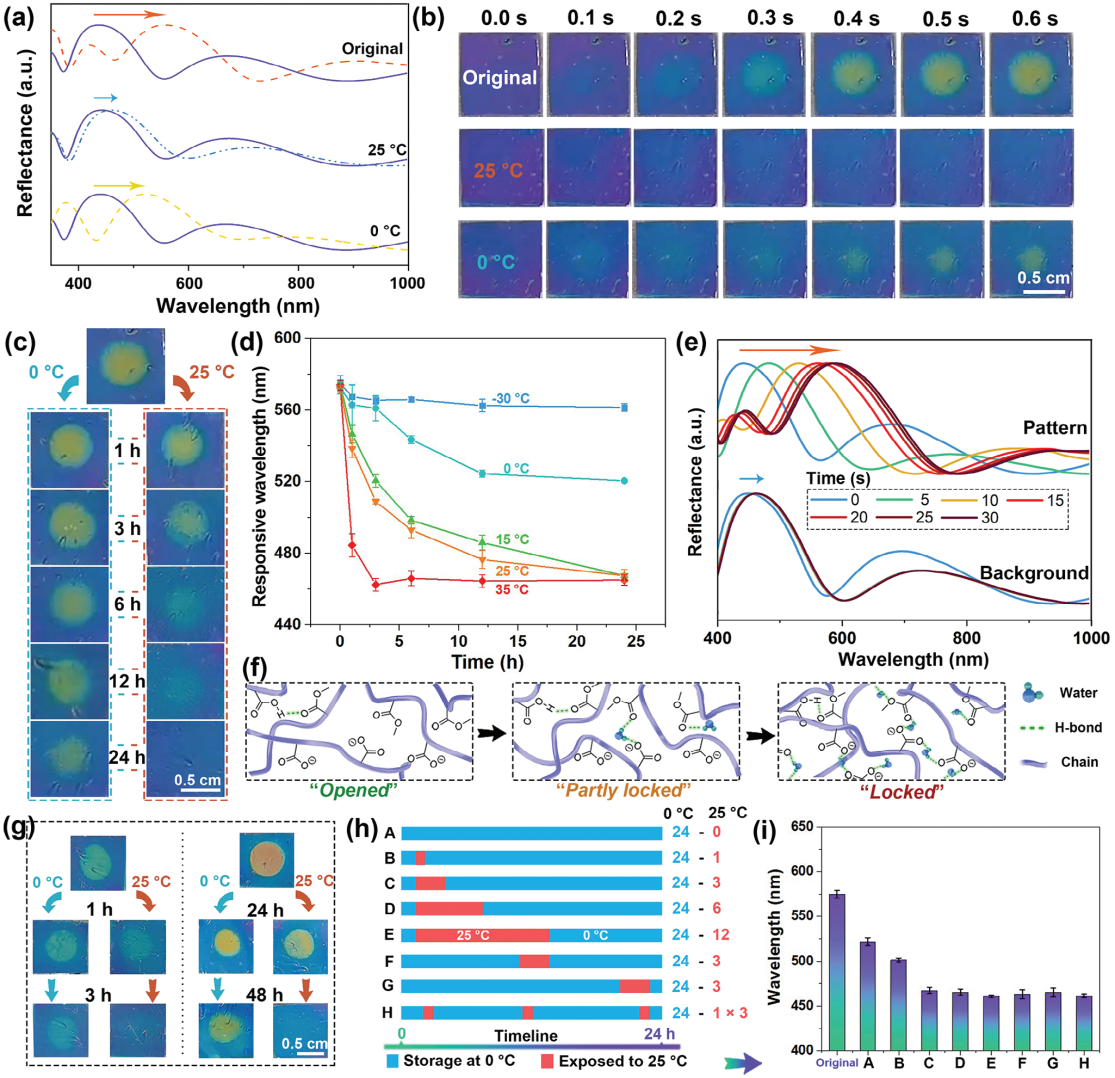
Variations of a) reflection spectra and b) photographs of the Na_2_CO_3_‐treated 1DPC exposed to water vapor before and after being stored at 0 and 25 °C for 24 h. c) Photographs of the Na_2_CO_3_‐treated 1DPC exposed to water vapor after various time at 0 and 25 °C. d) Variations of reflection wavelengths of the Na_2_CO_3_‐treated 1DPC exposed to water vapor after being stored at different temperatures for various time. e) Reflection spectra of the pattern and the background areas of the unresponsive 1DPC in 0.1 m NaHCO_3_ solutions. f) Schematic diagrams of the hydrogen bond formation process of the copolymer. g) Photographs of different Na_2_CO_3_‐treated 1DPCs exposed to water vapor after various time at 0 and 25 °C. h) Schematic diagrams and i) wavelengths of the Na_2_CO_3_‐treated 1DPCs exposed to water vapor after 24 h at 0 and 25 °C.

Typically, a violet 1DPC with a stopband of 445 nm was utilized for TTI fabrication. The center of the photonic film underwent treatment with a 0.1 m Na_2_CO_3_ solution for 60 s. Upon exposure to water vapor, the wavelength of the central region shifted to 575 nm in 0.6 s, revealing an orange–yellow pattern (**Figure** **4**a,b). However, after storage at room temperature (25 °C) for 24 h, no obvious wavelength shifts or patterns were evident under water vapor stimulation. In contrast, moisture induced a reflected peak shift from 445 to 525 nm, generating a green pattern on the TTI after 24 h storage at 0 °C. Figure 4c,d illustrates after storing the photonic film at 0 °C for 1, 3, 6, 12, and 24 h, the responsive wavelength of the central pattern varied from the original 573 to 563, 561, 544, 524, and 520 nm, respectively. Whereas, the wavelengths were 539, 509, 493, 478, and 465 nm under water vapor after storage at 25 °C for 1, 3, 6, 12, and 24 h, respectively. Under −30 °C storage, the wavelength of the responsive pattern is located at ≈570 nm toward water vapor, the responsiveness almost unchanged even after 24 h storage. Conversely, after storage at 35 °C for 3 h, the water vapor only induced a wavelength shift of 8 nm from 455 to 463 nm. In brief, the indicators exhibit robust responsiveness to water vapor after being stored in low‐temperature environments. Nevertheless, when exposed to elevated temperatures, their responsiveness exhibited an irreversible decline over time, with the rate of performance degradation escalating proportionally to higher storage temperatures.

Remarkably, the diminished responsiveness of the TTIs can be restored through immersion in 0.1 m NaHCO_3_ aqueous solutions. Figure 4e shows the wavelength of the unresponsive pattern underwent a red shift from 445 to 590 nm, while the wavelength of the background shifted only to 465 nm. This resulted in an orange–yellow pattern with a consistently blue background (Figure S8, Supporting Information). Consequently, the TTIs regained the initial responsiveness, presenting an orange‐yellow pattern in the presence of water vapor (Figure S9, Supporting Information). Additionally, cyclic experiments provide evidence of the stable regeneration process for the TTIs. (Figure S10, Supporting Information).

As Figure 4f shows, the conversion of ─COOH to ─COO^−^ hampered the hydrogen bond formation within the copolymer, leaving it in an open state. Consequently, swift water absorption and swelling occurred upon exposure to water vapor from human breath, causing distinct wavelength and color changes. Remarkably, with the extension of time, an increasing number of ambient water molecules are progressively adsorbed into the polymer network and reach equilibrium at room temperature, manifesting a dynamic moisture adsorption process. The gradual diffusion and adsorption of ambient water molecules established hydrogen bonding connections between copolymer chains, locking the copolymer network into a self‐shielded state. This hindered the rapid penetration and diffusion of external water molecules, resulting in reduced moisture responsiveness in 1DPC. The establishment of this self‐shield state can be efficiently restrained in a low‐temperature environment by decelerating or interrupting the motion of copolymer chains and water molecules. When exposed to 0.1 m NaHCO_3_, the hydrogen bonds in the copolymer were destroyed, and the 1DPC regained water vapor‐induced color‐changing performance. While the highly dilute alkaline conditions limited carboxyl reactivity, impeding new ─COO^−^ formation. Consequently, the background retained the original low water vapor responsiveness.

Notably, the 1DPCs, exhibiting diverse responsive colors toward water vapor, can serve various time interval requirements. For instance, in the scenario of products requiring a refrigerated storage temperature of 0 °C, the indicator with a final orange–yellow pattern (described in Figure 4c) is suitable for 24 h monitoring at an environmental temperature of 25 °C. Figure 4g shows a 1DPC treated by 0.1 m Na_2_CO_3_ for 30 s exhibiting a green color under water vapor stimulation. After storage at 25 °C for 3 h, the pattern became indiscernible under water vapor exposure. Conversely, the 1DPC stored at 0 °C maintained a clear pattern to water vapor. Thus, it can be employed for short‐term (3 h) temperature indicating. For long‐term temperature monitoring, a photonic film with a red pattern toward water vapor was obtained by treatment with 0.1 m Na_2_CO_3_ for 90 s. After 48 h of storage at 25 °C, the indicator lost responsiveness to water vapor. In contrast, the 1DPC stored at 0 °C maintained excellent color‐changing properties in response to water vapor, making it suitable for monitoring items in cold storage for a period of 48 h.

To summarize the above, the copolymer P(MMA‐AA) treated with low‐intensity (low concentration, short duration) Na_2_CO_3_ solution contains a small amount of carboxylate anions. A limited number of water molecules adsorbed in a short period can lock its network via hydrogen bonds, resulting in a loss of responsiveness of the 1DPC to water vapor stimulation. Therefore, the TTI can be employed for short‐term cold chain monitoring. In contrast, the copolymer network treated with high‐intensity (high concentration, long duration) Na_2_CO_3_ solution exhibits a significant increase in carboxylate anions. A longer duration is required to adsorb more water molecules and lock its network, causing the TTI to lose responsiveness to external water vapor stimulation. Consequently, it is suitable for long‐term storage monitoring. This principle allows the customization of TTIs to meet specific practical requirements.

In practical cold chain storage scenarios, products may undergo abrupt temperature elevations, impacting their final quality. We conducted simulation experiments involving a TTI placed in 0 °C cold storage for 24 h, interrupted by exposure to a 25 °C environment at varying intervals (Figure 4h). Figure 4i illustrates after 24 h at 0 °C, the 1DPC exhibited a reflected peak at 525 nm in response to water vapor, presenting a green pattern. During this 24 h refrigerated period, subjecting the 1DPC to a 25 °C environment for 1 h caused a blue shift of the moisture‐responsive wavelength to 500 nm, resulting in a blue‐green pattern. Exposing the indicator to a 25 °C environment for >3 h shifted the wavelength to ≈465 nm, making the pattern invisible. Therefore, the 1DPCs served as effective time‐temperature indicators for monitoring the quality of products during the cold chain storage.

### 2.4 Self‐Shielded Mechanism of Moisture‐Responsiveness

**Figure 5:**
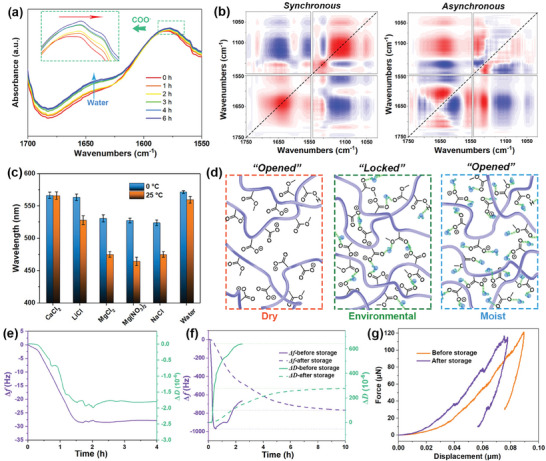
a) Time‐dependent FTIR adsorption spectra of Na_2_CO_3_‐treated P(MMA‐AA) after being stored at 25 °C and 53% RH for varying durations. b) 2D correlation synchronous and asynchronous spectra generated from (a). c) Wavelengths of the indicator exposed to water vapor after being stored at 0, 25 °C, and various RH for 24 h. d) Schematic diagrams of Na_2_CO_3_‐treated P(MMA‐AA) in dry, environmental, and moist conditions. Frequency and dissipation changes of Na_2_CO_3_‐treated P(MMA‐AA) film e) at 25 °C and 53% RH for varying durations, f) before and after being stored at 25 °C and 53% RH for 24 h. g) Force variations when the force sensor pressed the Na_2_CO_3_‐treated P(MMA‐AA) film before and after being stored at 25 °C and 53% RH for 24 h.

The foregoing has mentioned that the diminished water vapor responsiveness of the indicator is primarily attributed to the crosslinking effect within the copolymer arising from the penetration of ambient water molecules and the subsequent formation of hydrogen bonds. To investigate the hydrogen bond formation process and the interactions between water molecules and groups, time‐dependent infrared spectra and 2D correlation spectra of the copolymers during storage at room temperature were studied. Over time, the intensity of the characteristic absorption peak at 1640 cm^−1^ attributed to water molecules gradually increased, providing evidence that during the storage process of the copolymer at room temperature, there was progressive adsorption of water molecules from the environment. Simultaneously, the shift of the ─COO^−^ peak at 1570 cm^−1^ toward lower wavenumbers signified the formation of hydrogen bonds between carboxylate ions and water molecules (**Figure** **5**a).

To further investigate the binding dynamics between each functional group and water molecules, we analyzed 2D correlation spectra of the copolymer derived from time‐dependent FTIR spectra, specifically focusing on the regions of *ν*(C═O) and *ν*(C─O─C) (Figure 5b) during extended room‐temperature storage period (25 °C, 53% RH). Notably, peaks at ≈1736 and 1695 cm^−1^ were identified, corresponding to C═O groups. Simultaneously, the appearance of split peaks around 1565 and 1552 cm^−1^ was attributed to COO^−^ groups. Additionally, several subtle peaks associated with C─O─C groups emerged at ≈1150, 1105, and 1050 cm^−1^. Following Noda's judging rule,^[^ ^24^ ^]^ the sequential evolution of functional groups during extended periods of storage can be succinctly outlined as follows: 1736 → 1050 → 1105 → 1150 → 1565 → 1552 → 1640 → 1695 cm^−1^ (→ means earlier than). This sequence suggests the ordering of hydrogen bonding formation between groups and water molecules: *ν*(C═O) (ester group) → *ν*(C─O─C) (ester group) → *ν*(COO^−^) → *δ*(O─H) (water) → *ν*(C═O) (carboxyl group). That is, upon infiltration of water molecules from the environment into the copolymer interior, the hydrogen bond is initially established with ester groups, followed by carboxylate ions, and ultimately with carboxyl groups within the copolymer.

It is hypothesized that the environmental water molecules play a crucial role in attenuating the responsiveness of the 1DPC to water vapor stimulation. To validate this, comparative experiments were conducted in different humidity at 0 and 25 °C (as representative temperature points). Specifically, conditions of 0% and 100% relative humidity (RH) were achieved using anhydrous CaCl_2_ and water, respectively. Humidity levels of 11%, 33%, 53%, and 75% at 25 °C were established using saturated LiCl, MgCl_2_, Mg(NO_3_)_2_, and NaCl aqueous solutions. Figure 5c illustrates that, regardless of storage temperature, the 1DPC maintained its original responsiveness to water vapor with a responsive wavelength of ≈575 nm after 24 h under both dry and 100% RH conditions. However, when exposed to humidity levels between 33% and 75% for 24 h, the responsive wavelength of 1DPC to water vapor decreased. Additionally, the moisture‐responsiveness of the 1DPC after storage in atmospheres with 33−75% humidity remained relatively consistent. This humidity range aligns with common environmental conditions, demonstrating the applicability of this TTI for monitoring time‐temperature conditions in natural settings.

In a dry atmosphere, without the participation of ambient water molecules, carboxylate ions within the copolymer struggle to form effective hydrogen bonds (Figure 5d). Even after prolonged storage, significant swelling can occur upon exposure to water vapor, inducing noticeable color changes in 1DPC. However, when stored in a natural environment, 1DPC underwent hydrogen bond‐induced cross‐linking between ambient water molecules and copolymer groups, impeding immediate responsiveness to external water vapor stimuli. Under 100% humidity, a large number of water molecules rapidly infiltrated the copolymer network, causing nearly all carboxylate ions to individually bond with water molecules through hydrogen bonds. This also prevented the effective formation of hydrogen bonds among copolymer groups, leaving the copolymer in an “unlocked” state. Therefore, even after prolonged storage, the 1DPC still can generate significant wavelength and color changes in response to water vapor.

To validate this hypothesis, QCM‐D was employed to monitor the frequency and dissipation changes of the Na_2_CO_3_‐treated copolymer during prolonged storage at 25 °C and 53% RH. As depicted in Figure 5e, the frequency decreased to ‐28 Hz after 1.5 h, concomitant with a decrease in dissipation to −1.6 × 10^−6^. This frequency decrease reflects a gradual increase in the mass of the copolymer film, confirming the continuous water adsorption. The diminished dissipation indicates a decrease in the film's viscoelasticity, suggesting heightened rigidity and substantiating the crosslinking influence of water molecules on the copolymer network. Moreover, the Na_2_CO_3_‐treated copolymer film exhibited a maximum frequency reduction of 980 Hz within 0.5 h under water vapor stimulation, with dissipation increasing to 640 × 10^−6^ within 3 h (Figure 5f). But following storage at 25 °C and 53% RH, the frequency reduction under water vapor was 780 Hz after 10 h, and the dissipation increased to 280 × 10^−6^. This indicates a notable attenuation in the copolymer's responsiveness and swelling tendency to water vapor.

Finally, the reduced modulus of the Na_2_CO_3_‐treated copolymer before and after storage was characterized using the micro‐nano mechanical testing system. Under the same vertical displacement of the sensing probe relative to the film surface, a higher force was measured for the copolymer after storage at 25 °C and 53% RH for 24 h (Figure 5g). The modulus of the copolymer film, measured after storage, increased from the initial 15.28 to 16.15 MPa. This further substantiated the cross‐linking effect of water molecules on the copolymer network.

### 2.5 Applications of 1DPC‐Based TTIs

**Figure 6:**
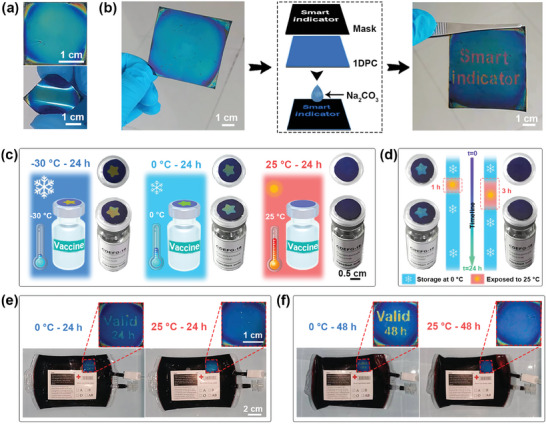
a) Photographs of a 1DPC assembled on the PET film. b) A photograph of a larger‐sized 1DPC, a schematic diagram of the preparation process for a patterned 1DPC, and a photograph of the patterned 1DPC exposed to water vapor. c) Photographs of the indicators on vaccine vials exposed to water vapor before and after being stored at −30, 0, and 25 °C for 24 h. d) Photographs of the indicators on vaccine vials exposed to water vapor after storage at 0 °C for 24 h with an additional exposure to a 25 °C atmosphere for 1 and 3 h. Photographs of the indicators on blood bags exposed to water vapor before and after being stored at 0 and 25 °C for e) 24 h and f) 48 h.

For practical applications, 1DPCs with diverse and brilliant colors were prepared on flexible black PET films (**Figure** **6**a; Figure S11, Supporting Information). These 1DPCs stably adhered to the PET films, and exhibited flexibility without cracking or detachment (Figure S12, Supporting Information). Additionally, a larger‐sized photonic film (6 cm × 6 cm) was successfully fabricated (Figure 6b). Employing a mask and treating with Na_2_CO_3_ solutions, responsive patterns, and texts could be printed on the films. Under moisture stimulation, the text “Smart indicator” on the 1DPCs became visible, exhibiting a red color.

To assess the practical utility, star‐patterned 1DPC indicators with orange‐yellow responsive color were applied to vaccines to simulate cold transportation scenarios. As depicted in Figure 6c, the indicator on the vaccine vial presented the original orange–yellow indicating pattern toward moisture stimulation after 24 h storage at −30 °C. After 24 h storage at 0 °C, the indicator displayed a green star‐shaped pattern in response to water vapor. However, following storage at 25 °C, the star‐shaped indicating pattern ceased to appear toward water vapor, signaling a loss of vaccine efficacy due to prolonged exposure to elevated temperatures. As depicted in Figure 6d, throughout the 24‐h cold chain storage at 0 °C, exposure to a 25 °C environment for 1 h caused the indicator on the vaccine vial to display a blue pattern. After 3 h of exposure to the same environment, the indicator showed no pattern in response to moisture, indicating prolonged exposure to high temperatures and the consequential loss of vaccine efficacy.

Similarly, the indicator with an orange–yellow responsive pattern attached to the blood bag exhibited a green “Valid 24 h” signal under water vapor after 24 h of storage at 0 °C, confirming the blood's validity (Figure 6e). However, after 24 h at 25 °C, the TTI on the blood bag showed no information toward water vapor, indicating blood deterioration. Furthermore, an indicator featuring a red “Valid 48 h” responsive pattern was created for extended temperature monitoring. In Figure 6f, after 48 h at 0 °C, the indicator on the blood bag showed a yellow signal upon exposure to water vapor. However, at 25 °C after the same storage period, no visible response was observed when subjected to water vapor. These experimental results confirmed the favorable practical application of the TTI.

According to the aforementioned principles, covering a hydrophobic polydimethylsiloxane (PDMS) film onto the indicator's surface can effectively isolate it from ambient moisture, preventing water molecule absorption and maintaining moisture‐induced color‐changing performance. For instance, when stored at room temperature for 3 days under PDMS protection, the indicator exhibited sustained moisture responsiveness without noticeable alterations (Figure S13, Supporting Information). Therefore, by using this strategy, pre‐use indicators can be reliably preserved for an extended duration at room temperature, presenting a notable advantage over their counterparts.

Additionally, the moisture‐responsive patterns on the indicators can also be temporarily concealed through repeated deionized water washing, and reactivated by 0.1 m NaHCO_3_ stimulation, restoring moisture‐stimulated color‐changing ability. This characteristic positions the indicator as a versatile anti‐counterfeiting label (Figure S14, Supporting Information). For instance, when applied to vaccines, the TTI served both as a quality indicator and an anti‐counterfeiting measure (Figure S15, Supporting Information). In the preceding discussion, the 1DPC consistently retained its responsiveness to water vapor, regardless of storage temperature in a dry atmosphere. This property makes it a reliable indicator for assessing the efficacy of some moisture‐susceptible materials or reagents during their storage in dry conditions (Figure S16, Supporting Information).

## 3 Conclusion

In summary, we demonstrate a novel moisture‐responsive 1DPC time‐temperature indicator with hydrogen bond‐mediated self‐shielded structural color which can be used to indicate and monitor the quality and efficacy of perishable products in the cold chain logistics. The TTI can be custom‐tailored based on the duration and temperature requirements according to the formulation storage and transportation processes. Additionally, the indicator possesses renewability, making it reusable. Significantly, the 1DPC indicator can be constructed on the flexible PET film, with designed responsive patterns, allowing it to be conveniently attached to the detection target as an indicator label. The moisture‐responsive pattern of the TTI can be reversibly concealed and revealed in an easy manner, enhancing its role as a smart anti‐counterfeit label. It also serves as an indicator to assess moisture absorption in hygroscopic reagents during dry storage, ensuring their efficacy.

## 4 Experimental Section

### Chemicals and Materials

MMA and AA were purchased from Tianjin Bodie Chemical Co., Ltd. Surfactant cetyl trimethyl ammonium bromide (CTAB) and initiator potassium persulfate (KPS) were supplied by Tianjin Kemio Chemical Reagent Co., Ltd and Xilong Chemical Co., Ltd, respectively. Nano TiO_2_ power and silicon wafers were purchased from Xuancheng Jingrui New Material Company and Zhejiang Lijing Silicon Material Company. Sylgard 184 silicone elastomer kit was obtained from Dow Chemical Company. PET films were purchased from an online store. The liquid contained in the vial is deionized water, employed for simulating vaccines. Blood utilized in the research is synthetic blood designed for film and television production, procured from an online store.

### Preparation of P(MMA‐AA)

Copolymer nanoparticles were synthesized via a modified microemulsion polymerization. 10.0 g MMA and 1.0 g AA were first mixed uniformly. Then 2.0 g of the above‐mixed monomers, 0.67 g CTAB, and 50.0 mL deionized water were added to a 150 mL flask and the mixture was heated to 75 °C for 20 min under nitrogen. Then 33.0 mg KPS dissolved in 5.0 mL deionized water was added into the above flask. The polymerization proceeded for 30 min. After that, the remainder of the mixed monomers were added dropwise to the polymerizing system within 1.0 h. Then the reaction was kept at 75 °C for another 30 min. When the polymerization was finished, the product was cooled down to room temperature. Finally, the prepared P(MMA‐AA) microemulsion was diluted to the appropriate concentration with deionized water before use. By changing the monomer ratio and combining it with the optical phenomena of the prepared 1DPCs, an optimal monomer composition was identified. According to the integral area ratio in the ^1^H NMR spectrum (Figure S2, Supporting Information), the ratio of PMMA to PAA components in the copolymer is ≈12:1.

### Assembly of P(MMA‐AA)/TiO_2_ 1DPC

1DPCs were prepared by alternately spin‐coating the copolymer microemulsion (1.0 to 5.0 wt.%) and TiO_2_ dispersion (1.0 to 3.0 wt.%) onto silicon wafers and PET films by using a spin coater (Laurell WS‐650SZ‐6NPP/LITE). TiO_2_ aqueous dispersion was made by dispersing a certain amount of TiO_2_ power in deionized water. The copolymer layer was the first layer just next to the substrates, and the TiO_2_ layer was the top layer on the surface of the film during the whole preparation process. The layers were dried at 60 °C for 60 s after each assembled layer. In efforts to enhance structural stability, the 1DPCs underwent exposure to saturated ethanol vapors followed by heating at 100 °C for 5 min. The initially stacked P(MMA‐AA) nanoparticle layer exhibited numerous gaps between particles (Figure S2, Supporting Information). Through the ethanol vapor‐triggered swelling and deswelling process, the P(MMA‐AA) nanoparticles established interconnections, forming an integral film that effectively bolstered the structural stability of the 1DPC (Figure S4, Supporting Information). Meanwhile, this process led to a reduction in the thickness of the P(MMA‐AA) layer.

### Fabrication of TTIs

For the fabrication of TTIs, Na_2_CO_3_ aqueous solutions with different concentrations were added onto the surface of the 1DPCs for a diverse time. After that, the alkaline solutions were washed off by deionized water. Then the photonic films were dried and used as TTIs. To prepare a responsive pattern, patterned hollow masks were pre‐attached to the surface of 1DPC, followed by the dropwise addition of Na_2_CO_3_ aqueous solutions onto the mask surfaces. The TTIs can be protected by PDMS films for prolonged storage at room temperatures. The PDMS films were prepared by heating the mixture of oligomer and cross‐linker at 80 °C for 2 h.

### Characterization

^13^C NMR and ^1^H NMR spectra of the copolymers were characterized by the NMR technique (Bruker AVANCE III 500, Bruker, Switzerland). FTIR spectra of P(MMA‐AA) before and after treatment of Na_2_CO_3_ aqueous solution were measured on a spectrometer (6700, ThermoFisher, the United States). The cross‐sectional morphologies of the 1DPC were recorded by using an ultra‐high resolution scanning electron microscope (SU8220, Hitachi, Japan). The morphologies and thickness differences of the photonic films were recorded by using an atomic force microscope (JPK Nanowizard 4XP, Bruker, Germany). The reflection spectra of the photonic films were measured by using a fiber optic spectrometer (QE Pro, Ocean Insight, the United States). Optical microscope images were captured by utilizing a digital microscope (VHX‐7000N, Keyence, Japan). The force changes of the P(MMA‐AA) film under water vapor stimuli and reduced modulus before and after treatment of Na_2_CO_3_ solutions were measured by using a micromechanical testing and assembly system (FT‐MTA03, FemtoTools, Swiss). The variations of frequency and dissipation of the photonic films were monitored utilizing the QCM‐D sensor (Q‐Sense E1, Biolin Scientific, Sweden). The changes in frequency and energy dissipation reflect alterations in mass loading and viscoelastic properties respectively of the films deposited on quartz crystal sensors. A reduction in frequency is expected as the increase of mass which indicates material adhered to the QCM‐D sensor undergoes molecular adsorption. Simultaneously, an increase in the dissipation value signifies a decrease in the robustness of the adsorbed layer, indirectly suggesting potential swelling of the assembled layers.

## Conflict of Interest

The authors declare no conflict of interest.

## Supporting information

Supporting Information

## Data Availability

The data that support the findings of this study are available from the corresponding author upon reasonable request.
