# Supplementary material for: Hydrogen Bond‐Mediated Self‐Shielded Moisture‐Responsive Structural Color for Time‐Temperature Indicating
Source: Adv Sci (Weinh). 2024 Feb 26;11(17):2310060. doi: 10.1002/advs.202310060 (PMC11077668; doi:10.1002/advs.202310060)
Supplement: Supplementary file 1 — Supporting Information [file ADVS-11-2310060-s001.pdf]

ADVANCED SCIENCE
Open Access

Supporting Information

for *Adv. Sci.*, DOI 10.1002/advs.202310060

Hydrogen Bond-Mediated Self-Shielded Moisture-Responsive Structural Color for Time-Temperature Indicating

*Donghui Kou, Lei Gao, Ruicheng Lin, Shufen Zhang and Wei Ma**

Supporting Information

Hydrogen Bond-Mediated Self-Shielded Moisture-Responsive Structural Color for Time-Temperature Indicating

*Donghui Kou, Lei Gao, RuiCheng Lin, Shufen Zhang, Wei Ma**

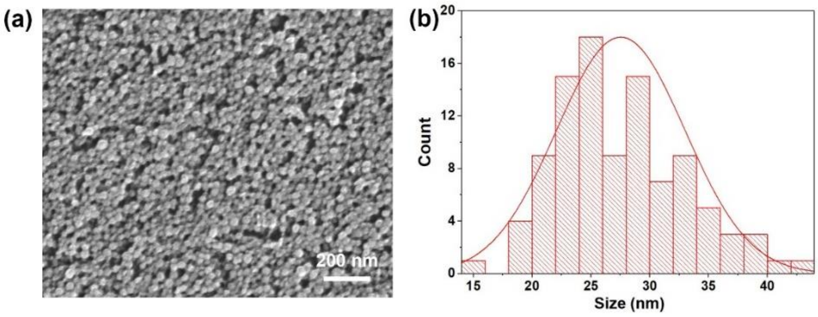

Figure S1. SEM image and particle size distribution of P(MMA-AA) nanoparticles.

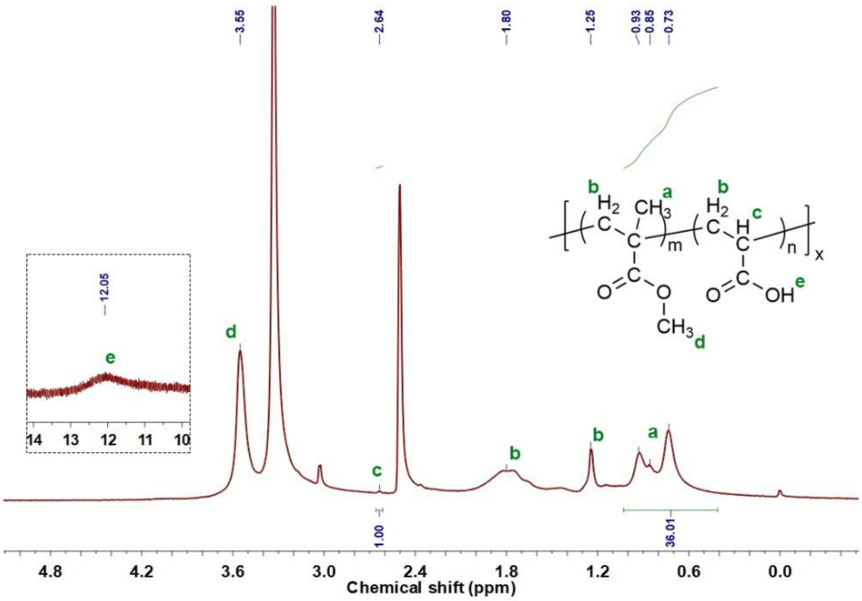

Figure S2. ^{1}H NMR spectrum of P(MMA-AA) in DMSO-d_6 solution.

^{1}H NMR (500 MHz, ppm, DMSO-d_6): δ 3.55 (-O-C$\underline{\mathbf{H}}_3$), δ 0.73-0.93 (-C-C$\underline{\mathbf{H}}_3$), δ 1.25-1.80 (-C$\underline{\mathbf{H}}_2$-), δ 2.64, (-(CH$_3$)C$\underline{\mathbf{H}}$-).

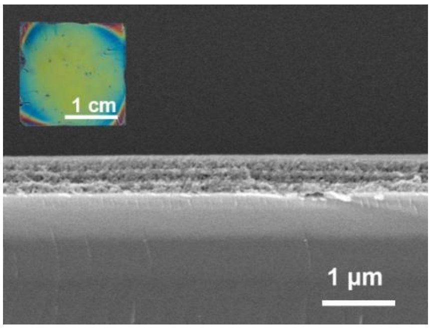

Figure S3. Photograph and cross-sectional SEM image of original P(MMA-AA)/TiO_2 1DPC. (The thicknesses of the copolymer and TiO_2 layers were measured to be 100 and 75 nm, respectively)

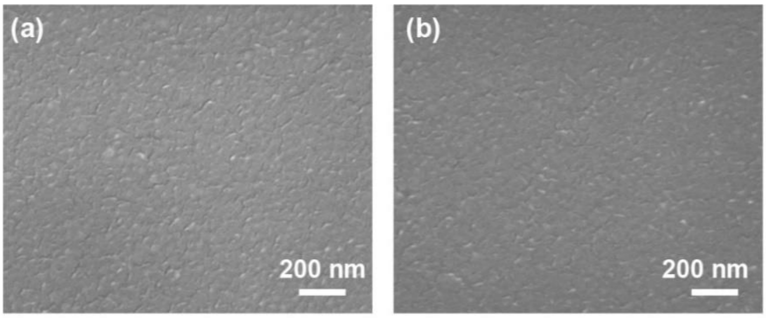

Figure S4. SEM images of (a) ethanol vapor and (b) heat treated P(MMA-AA) nanoparticle films.

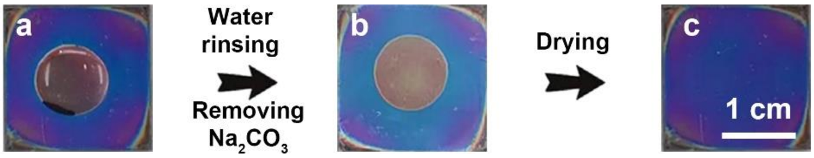

Figure S5. Photographs of the 1DPC treated by Na_2CO_3 solution and color change after water rinsing and drying.

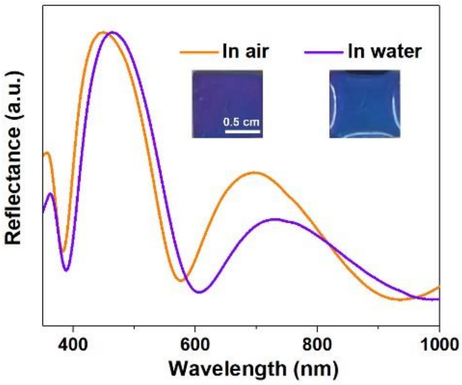

Figure S6. Photographs and reflection spectra of the untreated 1DPC in air and in water.

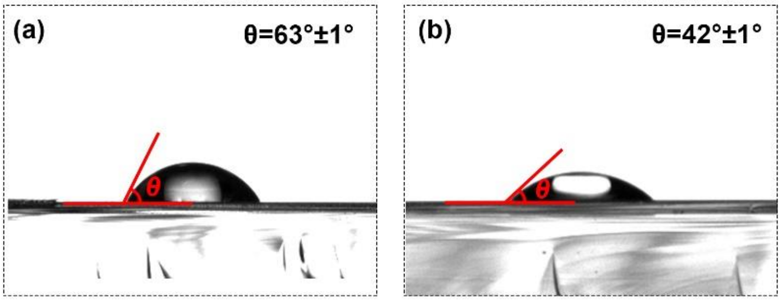

Figure S7. Water contact angles of (a) untreated and (b) Na_2CO_3-treated P(MMA-AA) film.

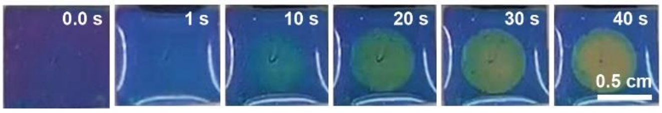

Figure S8. Photographs of an unresponsive indicators immersed in 0.1 M $NaHCO_3$ aqueous solutions.

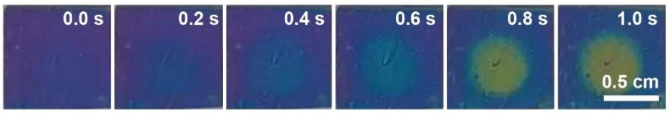

Figure S9. Photographs of a regenerated indicators upon exposed to water vapor.

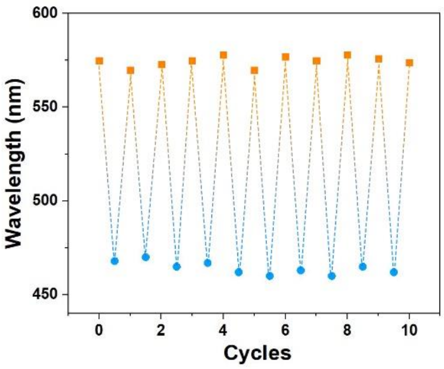

Figure S10. Photographs of a regenerated indicators upon exposed to water vapor. (The orange squares represent the wavelengths of the regenerated indicator in the presence of water vapor. The blue circles represent the wavelengths of the indicator exposed water vapor after storage at 35 ℃ for 3 hours.)

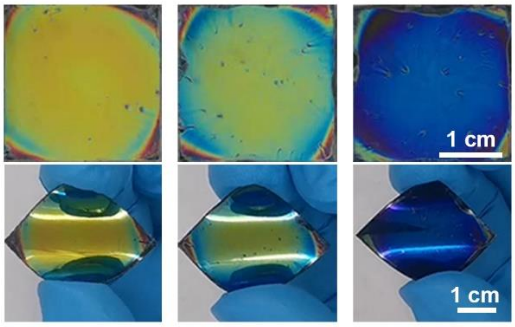

Figure S11. Photographs of the 1DPCs with various colors assembled on the PET films.

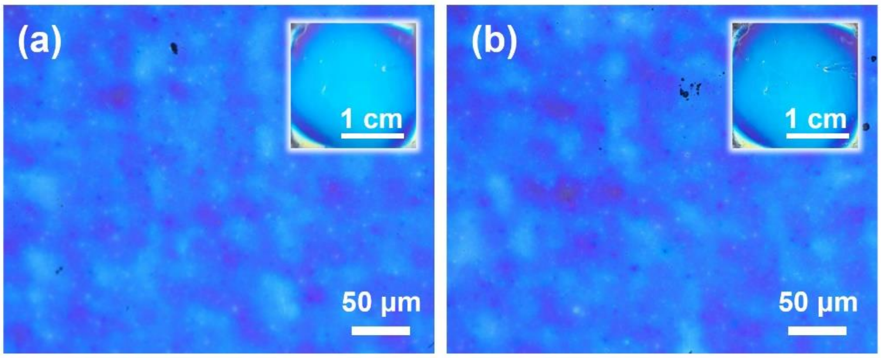

Figure S12. Optical microscope images and photographs (insets) of the TTI before and after 100 cycles of exposure to water vapor from human breath and temperatures ranging from 0 °C to 35 °C..

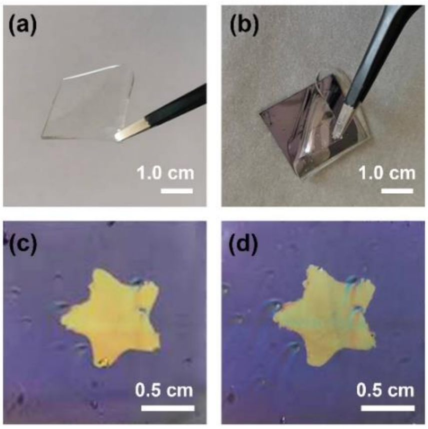

Figure S13. Photographs of (a) a PDMS film and (b) the TTI protected by the PDMS film. Photographs of the indicator upon exposure to water vapor (c) before and (d) after storage at 25 °C for 3 days with the protection of a PDMS film.

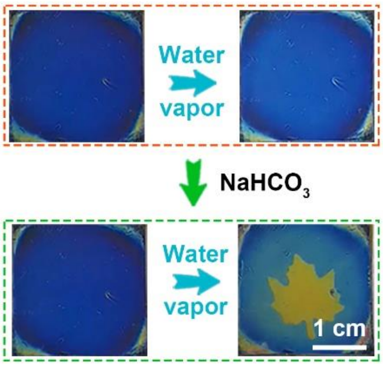

Figure S14. Photographs of the 1DPC with hidden pattern exposed to water vapor before and after treatment of 0.1 M $NaHCO_3$ solutions.

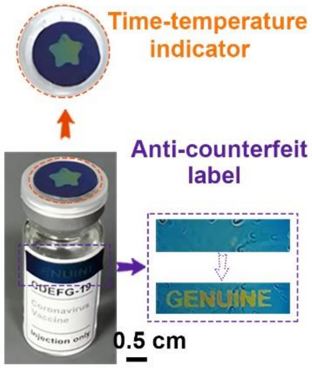

Figure S15. Photographs of the 1DPC attached on a vaccine vial with a hidden pattern exposed to water vapor before and after treatment of 0.1 M $NaHCO_3$ solutions.

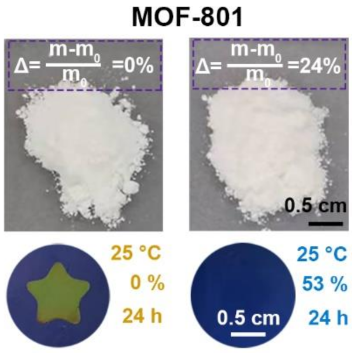

Figure S16. Photographs of MOF-801 powers and indicators exposed to water vapor after being stored at 0% and 53% RH for 24 h.

For the purpose of verifying this effectiveness and illustrating the indicative role of TTIs in the inconspicuous water absorption process of moisture-susceptible materials, MOF-801 was chosen as the model material which exhibits distinct water absorption characteristics without manifesting visually perceptible changes in its apparent morphology. As depicted in Figure S16,

after storing MOF-801 in a natural environment (53% RH) for 24 hours, the mass increased by 24% upon moisture absorption which is imperceptible to the naked eye. While the indicator under the same storage condition no longer exhibited a responsive pattern under water vapor stimuli, demonstrating the material has been exposed to a non-dry environment and absorbed moisture. In contrast, the indicator, accompanying the MOF-801 sealed in a dry environment, maintained its initial responsiveness to water vapor and exhibited an orange-yellow star pattern, indicating the material remains securely sealed in a dry environment and retains its efficacy. The relevant details have been added in the revised supporting information.
